# Exploring early DNA methylation alterations in type 1 diabetes: implications of glycemic control

**DOI:** 10.3389/fendo.2024.1416433

**Published:** 2024-06-05

**Authors:** Barbara Čugalj Kern, Jernej Kovač, Robert Šket, Tine Tesovnik, Barbara Jenko Bizjan, Julia Galhardo, Tadej Battelino, Nataša Bratina, Klemen Dovč

**Affiliations:** ^1^ University Children’s Hospital, University Medical Centre Ljubljana, Ljubljana, Slovenia; ^2^ Faculty of Medicine, University of Ljubljana, Ljubljana, Slovenia; ^3^ Paediatric Endocrinology and Diabetes Unit, Hospital de Dona Estefânia - Central Lisbon University Hospital Center, Lisbon, Portugal; ^4^ Lisbon Academic and Clinical Center, NOVA Medical School, Lisbon, Portugal

**Keywords:** type 1 diabetes, glycemic control, DNA methylation, diabetes-related complications, long-read sequencing

## Abstract

**Background:**

Prolonged hyperglycemia causes diabetes-related micro- and macrovascular complications, which combined represent a significant burden for individuals living with diabetes. The growing scope of evidence indicates that hyperglycemia affects the development of vascular complications through DNA methylation.

**Methods:**

A genome-wide differential DNA methylation analysis was performed on pooled peripheral blood DNA samples from individuals with type 1 diabetes (T1D) with direct DNA sequencing. Strict selection criteria were used to ensure two age- and sex-matched groups with no clinical signs of chronic complications according to persistent mean glycated hemoglobin (HbA1c) values over 5 years: HbA1c<7% (N=10) and HbA1c>8% (N=10).

**Results:**

Between the two groups, 8385 differentially methylated CpG sites, annotated to 1802 genes, were identified. Genes annotated to hypomethylated CpG sites were enriched in 48 signaling pathways. Further analysis of key CpG sites revealed four specific regions, two of which were hypermethylated and two hypomethylated, associated with long non-coding RNA and processed pseudogenes.

**Conclusions:**

Prolonged hyperglycemia in individuals with T1D, who have no clinical manifestation of diabetes-related complications, is associated with multiple differentially methylated CpG sites in crucial genes and pathways known to be linked to chronic complications in T1D.

## 1 Introduction

Type 1 diabetes (T1D) is a chronic autoimmune disease that involves the autoimmune destruction of insulin-secreting pancreatic β-cells, resulting in disturbed glucose regulation and overt hyperglycemia. Therefore, individuals with T1D require lifelong insulin replacement therapy (1). The long-term dysregulation of blood glucose levels in T1D can lead to micro- and macrovascular complications, which are the primary causes of diabetes-related morbidity and mortality. Complications of diabetes include diabetic retinopathy, neuropathy, kidney disease, and cardiovascular diseases (2).

The importance of regulating blood glucose levels was observed during the Diabetes Control and Complication Trial and its follow-up observational study Epidemiology of Diabetes Interventions and Complications. The benefits of intensive insulin treatment were demonstrated many years after the end of the study despite equalization of glycemic control between the studied groups. The phenomenon commonly referred to as metabolic memory is believed to involve several key factors, including chronic inflammation, oxidative stress, glycation of proteins, and epigenetic mechanisms (3, 4).

Epigenetic mechanisms, such as DNA methylation, histone modifications, and non-coding RNAs, play a crucial role in regulating gene expression by modifying DNA accessibility or chromatin structure. Environmental factors, such as nutrition, drugs, chemicals, stress, and infection, can impact epigenetic mechanisms, thus influencing various physiological and pathophysiological processes (5, 6). There is growing evidence linking late complications of T1D to epigenetic mechanisms (7). Prolonged hyperglycemia is associated with alterations in epigenetic marks that persist even after the introduction of a normoglycemic environment. Alterations in DNA methylation have been found in the blood and tissues of individuals with T1D with late complications (8). Alterations have been associated with several chronic complications (9–11). However, the association between glycemic control and genome-wide DNA methylation profile in blood samples from individuals with T1D without clinical signs of chronic complications remains unclear. This study aimed to evaluate the association between persistently dysregulated glycemic control and early DNA methylation alterations in individuals with T1D.

## 2 Methods

### 2.1 Participants

**Figure 1:**
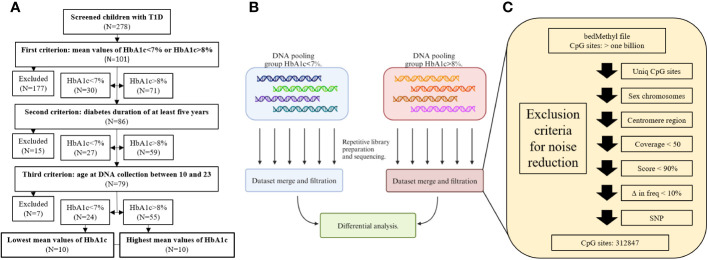
Flow charts of recruitment process **(A)**, library preparation and sequencing **(B)**, and filtration criteria for noise reduction **(C)**.

20 participants (11 males and 9 females) without clinical manifestations of chronic complications were recruited. Participants were selected from a National Childhood Registry for T1D, which contained records of 278 children with T1D, who were regularly managed at the Department of pediatric endocrinology, diabetes, and metabolic diseases outpatient clinic at the University Children Hospital Ljubljana, Slovenia. Participants were recruited according to selected study inclusion criteria and divided into two groups. Namely, calculated mean values of HbA1c persistently below 7% or above 8%, where mean values of HbA1c for the span of five years (three to four measurements per year) were used. This was followed by diabetes duration of at least five years and age at DNA collection between 10 and 23 years. Considering all criteria, 79 children qualified for the participation in the study, 24 children with mean values of HbA1c<7% and 55 children with mean values of HbA1c>8%. Among them, ten children with the lowest and highest mean values of HbA1c were selected into the final cohort for methylation analysis (**Figure 1A**). We hypothesize that analyzing participants from both extremes of mean HbA1c levels may reveal more apparent DNA methylation effects. Otherwise, uncovering significant differences in methylation might be obscured in analyses across the entire HbA1c range with an arbitrary threshold between groups, to reduce the effect of individual variability.

### 2.2 Samples and DNA methylation analysis

Participants’ peripheral blood samples were collected in EDTA tubes and DNA was isolated using the FlexiGene Isolation Kit (Qiagen, Cat# 51206) and stored at 4°C until further analysis. Since the participants’ samples were divided into two groups, one of 10 participants with HbA1c<7% and one of 10 participants with HbA1c>8%, for each group, the participants’ DNA was pooled together with equal mass. Therefore, each participant contributed ~10% to the pooled sample. Prior to pooling, DNA quantities were measured three times using the Qubit HS DNA Assay (Thermo Fisher Scientific, REF Q32854) and the mean value was used for pooling. Six libraries for the HbA1c<7% group and six libraries for HbA1c>8% group (twelve in total) were prepared and sequenced independently for each pooled sample (**Figure 1B**), to account for batch effect of library preparation and sequencing runs, for each pooled sample. Each pooled sample was sonicated to an average length of 3,000 bp to increase the number of active ends and improve the sequencing yield. Libraries were prepared using a Ligation Sequencing Kit (Oxford Nanopore Technologies, product code SQK-LSK109) according to the manufacturer’s protocol and sequenced separately on a PromethION flow cell (R9.4.1, Oxford Nanopore Technologies, product code FLO-PRO002).

### 2.3 Data preparation

All datasets generated from each sequencing run within the group were merged and analyzed as a single batch. Modified bases were called with Guppy basecaller (v6.3.8) using the high accuracy Remora model (dna_r9.4.1_450bps_modbases_5hmc_5mc_cg_hac) (12) and the human reference sequence GRCh38 (hg38) (13). The final BAM files were summarized to a bedMethyl file using the modbam2bed package (version 0.6.3.) (14). bedMethyl files underwent several filtering steps as shown in **Figure 1C**. First, regions with methylation calls were filtered for low quality data and prepared for the differential analysis between the two groups. Second, to avoid inaccurate differential methylation calls due to genomic structure and variation, only CpG sites present in both groups were considered for downstream analysis. Although there was no statistical difference in sex between the two groups, sex chromosomes were excluded from the analysis due to potential methylation differences in X chromosome inactivation. Centromeres were also excluded due to highly repetitive sequences. Next, to obtain the most accurate methylation call only CpG sites with coverage greater than 50 were included for further analysis. bedMethyl file score parameter provides information on how the calculated DNA methylation frequency is confounded by alternative calls and indicates the confidence or reliability of the calculated DNA methylation frequency. Only calls with a score greater than 900 were analyzed (14). Since the sequenced samples were pools of DNA, where each individual is estimated to contribute approximately 10% of the signal, all CpG sites with the difference in DNA methylation below 10% were omitted. In the case of a single nucleotide variant at the CpG sites identified by Clair3 (version v0.1-r12, model: r941_prom_sup_g5014), these CpG sites were excluded from the downstream analysis (15). Consequently, more than one billion CpG sites were reduced to the final 312,847 CpG sites, which were further analyzed.

### 2.4 Statistical analysis

To characterize the analyzed groups, the Wilcoxon signed-rank test and χ^2^-test were used. Differential methylation analysis was conducted using the R package DSS (version 2.46.0), which implements algorithms based on the dispersion shrinkage method followed by the Wald test to evaluate each CpG site. Smoothing was applied, which combined the information from nearby CpG sites to improve the estimation of methylation levels (16). A *p*<0.001 was used to consider statistically significant results. The false discovery rate (FDR) was calculated with the Bejamini-Hochberg procedure to correct for multiple comparisons and all differently methylated sites under selected *p-value* had FDR value below 0.05. Regions were formed by merging statistically significant neighboring CpG sites. The following restrictions were set *p*<1.0x10^–14^, a minimum number of CpG sites is equal or greater than 10, and a minimum region length of at least 100 bp. If the distance between two regions was less than 1500 bp, they were merged into one (16).

### 2.5 Annotation and pathway enrichment analysis

The R package annotatr (version 1.24.0) (17) was utilized to annotate differentially methylated CpG sites and regions genomic features. The CpG annotations source was obtained from the AnnotationHub package (18), the genetic annotations source was TxDb.Hsapiens.UCSC.hg38.knownGene (19), and the long-noncoding RNA source was obtained from the GENCODE database (https://www.gencodegenes.org). All plots were generated in R using the DSS, ggmanh (version 1.2.0) (20), and ggplot2 packages (version 3.4.1) (21). Signaling pathway enrichment analysis was performed using KEGG pathway over-representation analysis in the R package clusterProfiler (version 4.6.2). The KEGG database (https://www.kegg.jp) (22) was utilized for this purpose.

## 3 Results

### 3.1 Participants

At the time of blood sampling for DNA isolation, the participants had a median age of 16 years (interquartile range, IQR 13–21) and a median duration of T1D of 7 years (IQR 6–8). Participants were divided into two groups according to their average values of HbA1c (**Supplementary Table 1**) over a five-years period. Group characteristics are summarized in **Table 1**. No statistical differences in age, duration of T1D, or sex were observed between the groups.

**Table 1:** Group characteristics.

	HbA1c<7%	HbA1c>8%	*p-value*
**Number of participants** **Male and female (%)**	10 4 (40) and 6 (60)	10 7 (70) and 3 (30)	0.1775
**Median age** **IQR (years)**	13 11.25–21	16.5 16–19	0.3206
**Median duration of T1D** **IQR (years)**	7 6–7	7 6–8	0.613
**Median HbA1c** **IQR (%)**	6.44 6.36–6.46	9.97 9.75–10.34	

### 3.2 Identification of significantly differentially methylated sites and regions

**Figure 2:**
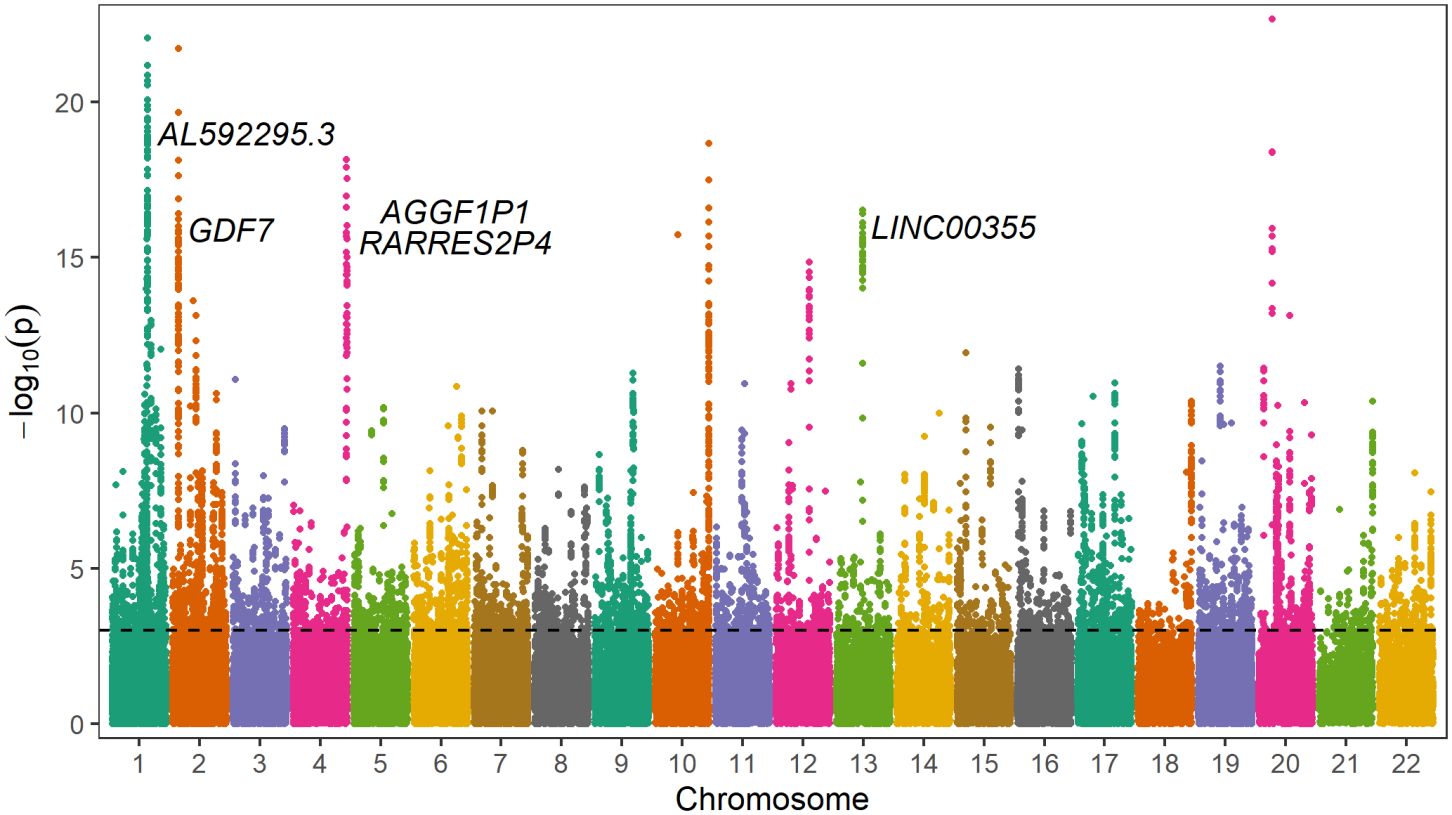
Manhattan plot of differentially methylated sites including gene annotations of differently methylated regions. The y-axis represents -log10(*p-value*) for association of each CpG site with different glycemic control regulation. The horizontal line represents the threshold for genome-wide significance (*p*<0.001, FDR<0.05).

On average 33.4 M (IQR 25.3–38.5 M) reads were generated per library, resulting in 66.8 Gb (IQR 55.0–84.9 Gb) of data. The average insert size per library was 3.6 kb (IQR 3.4–3.9 kb). After filtering the datasets and differential analysis, a total of 8,385 differentially methylated sites, 4,575 hypomethylated, and 3,810 hypermethylated in the group with HbA1c>8% compared to the group with HbA1c<7%, were identified (**Supplementary Table 2**). These CpG sites were annotated to 1,802 genes (**Supplementary Table 3**). Of these, 909 genes had hypomethylated sites, 840 genes had hypermethylated sites and 53 genes had both hypomethylated and hypermethylated sites. A Manhattan plot showing differentially methylated sites by chromosomal position is shown in **Figure 2**.

In the promoter regions or 1–5 kb upstream of the transcription start site, 270 genes showed hypermethylated sites, 264 genes showed hypomethylated sites, and 1 gene showed both types of methylation changes. Among them, hypermethylated sites with significant methylation difference (*p*<1.0x10^–10^) were annotated to *MRPS11P1, LOC107984012, LOC105373526, RNA5S6, RNA5S5, ATG16L2, GRB10, CLUAP1*, and C16orf90. Hypomethylated CpG sites with significant methylation difference (*p*<1.0x10^–10^) were annotated to the genes *LINC00355, CD1D, ELL2P1, RGPD2, KCNK15-AS1, CDIN1, LOC101928797, LINC00654*, mir-6080, *GXYLT1, PLAGL1, FLG-AS1, CCDC144NL, CHRNE*, and *LOC100996664*.

**Figure 3:**
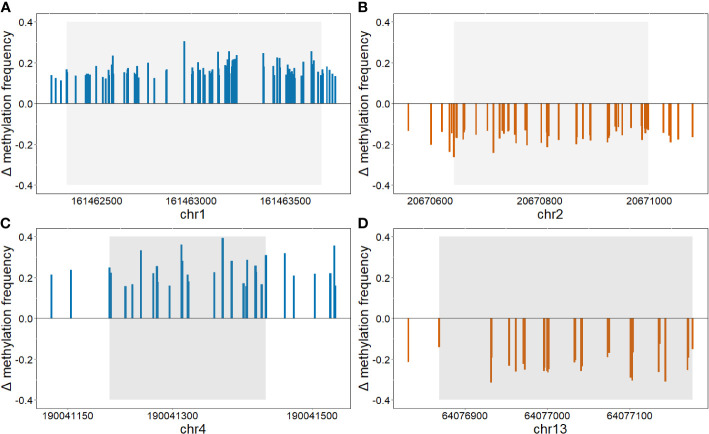
Differently methylated regions (threshold: *p*<1.0x10^–14^). Graphs show the difference in methylation frequencies between the two groups in regions: **(A)** chr1:161462342–161463692, **(B)** chr2:20670644–20670998, **(C)** chr4:190041210-190041434, and **(D)** chr13:64076869–64077176. Positive values show hypermethylation in group HbA1c>8%, whereas, negative values show hypomethylation.

Differentially methylated CpG sites with *p*<1.0x10^–14^ were further analyzed. A total of 188 differentially methylated sites were retained. The majority of these sites were in close proximity to each other and were therefore combined into differentially methylated regions. Finally, four regions were identified, two were hypermethylated and two were hypomethylated according to the HbA1c>8% group (**Table 2**, **Figure 3**). Hypermethylated regions were located in the intron of *AL592295.3*, a long non-coding RNA, and in the promoter region of *AGGF1P1* and 1–5 kb upstream region of *RARRES2P4*. Both are processed pseudogenes. Hypomethylated regions were found in exon 1 of *GFD7* gene and in the promoter region of *LINC00355*.

**Table 2:** Differently methylated regions (threshold: ***p*** <1.0x10^–14^).

chr	start	end	length	nCG	diff.Methy	gene	annotation
**chr1**	161462342	161463692	1351	91	-0.16	*AL592295.3*	intron
**chr2**	20670644	20670998	355	44	0.16	*GDF7*	exon
**chr4**	190041210	190041434	225	23	-0.23	*AGGF1P1* *RARRES2P4*	promotor 1–5 kb upstream
**chr13**	64076869	64077176	308	28	0.23	*LINC00355*	promotor

chr, chromosome; nCG, number of CpG sites; diff.Methy, the difference in methylation levels between two groups.

### 3.3 Genomic annotations of differentially methylated sites and functional enrichment analysis

**Figure 4:**
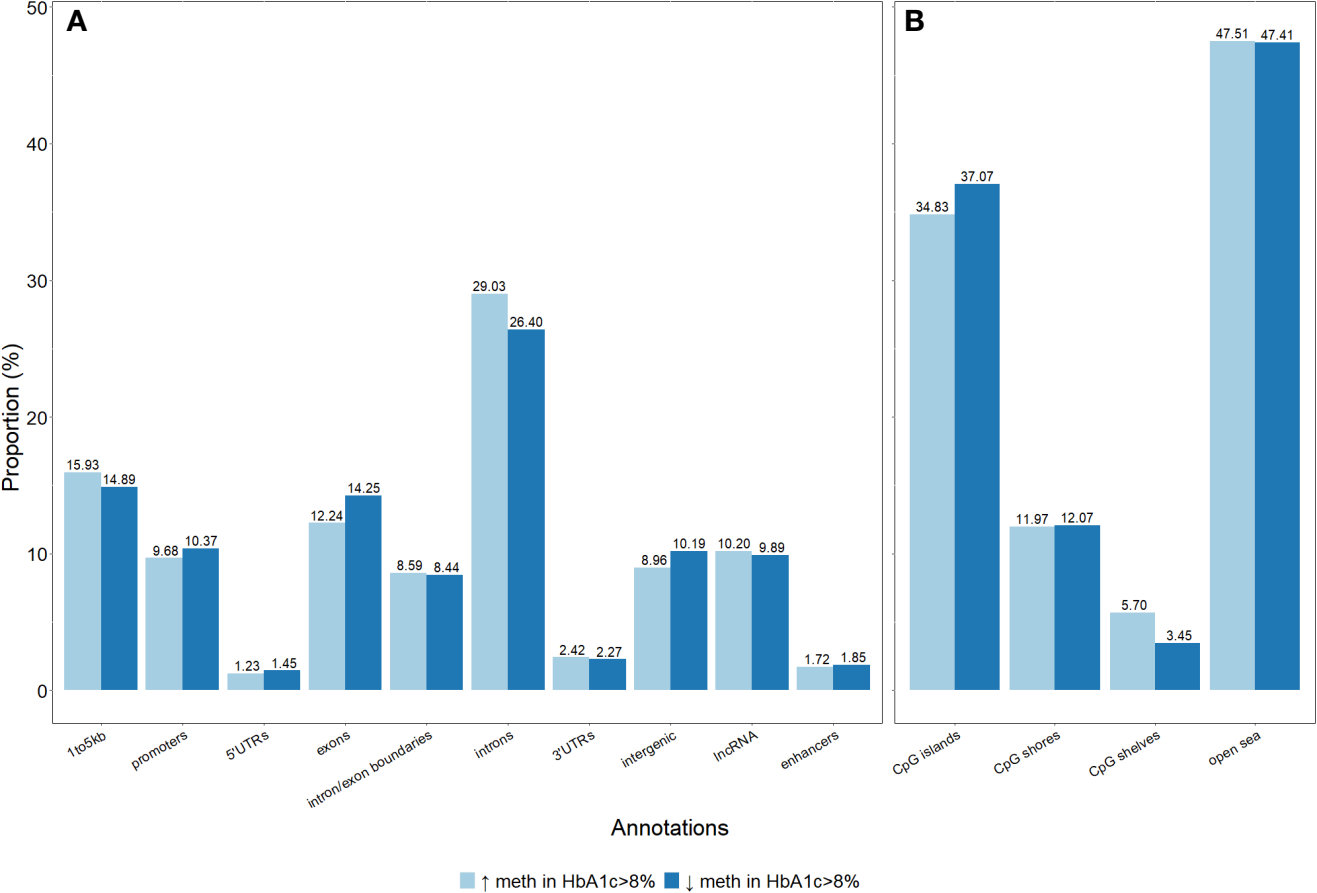
Distribution of genomic annotations of differentially methylated sites in the gene structures and CpG sites. **(A)** Differentially methylated sites per genic annotations. **(B)** Differentially methylated sites per CpG annotations.

The distribution of genomic annotations according to genomic features was not different between the two groups. Most of the differentially methylated sites were located in introns and open sea genomic positions (**Figure 4**).

**Figure 5:**
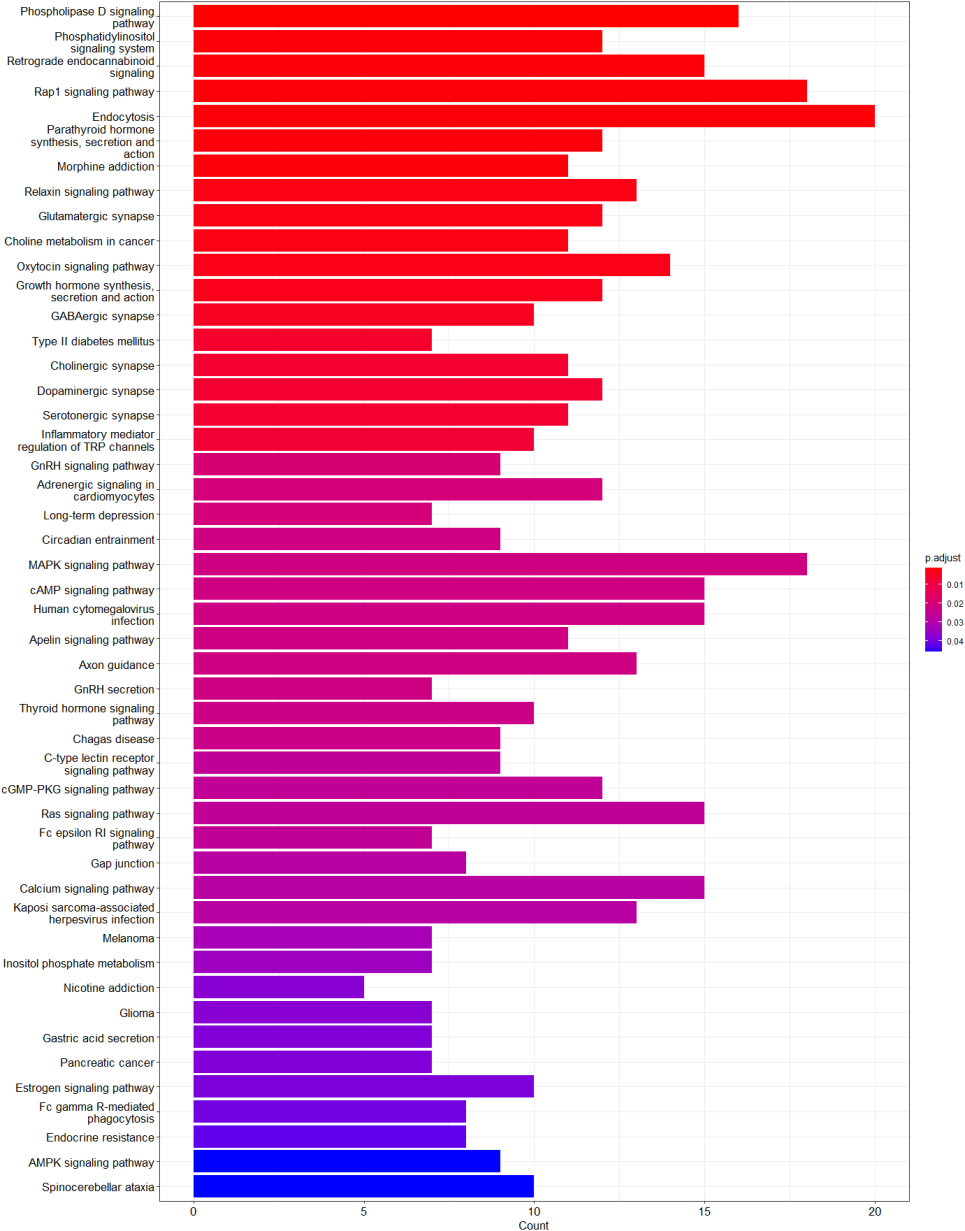
Enriched pathways of genes annotated to hypomethylated sites. Graph shows adjusted *p-value* and gene count for each enriched pathway. Red indicates the most significant enrichment and blue less significant while genes count is depicted as bar height.

The functional relevance of genes annotated at differentially methylated sites was analyzed by gene enrichment pathway analysis. No biological pathway was significantly enriched by the set of genes annotated to hypermethylated sites, whereas genes annotated to hypomethylated sites were significantly enriched in 48 pathways associated with differentially regulated glycemic control in participants with T1D (**Figure 5**). Among these pathways, the following genes were common: *ADCY4*, *CACNA1A*, *CACNA1C*, *EGFR*, *GNAI*, *GNAS*, *GNG4*, *GNG7*, *ITPR2*, *MAPK10*, *PDGFRA*, *PIK3CD*, *PIK3R1*, *PLCB1*, *PLCG2*, and *RAF1*. Among them, *ADCY4*, *GNAS*, *MAPK10*, *PIK3CD*, and *RAF1* genes had hypomethylated CpG sites located in promoter regions or 1–5 kb upstream of the transcription start site, while other genes had CpG sites were located in exons and introns.

## 4 Discussion

Epigenetic modifications represent a dynamic relationship between the genome and the environment (23). To the best of our knowledge, this is the first study to investigate the association of glycemic control with genome-wide differences in DNA methylation in blood samples from participants with T1D without evident clinical signs of chronic complications.

Our method of choice for detecting DNA methylation was novel, comprehensive direct nanopore DNA sequencing using long-read nanopore sequencing, which allows direct whole-genome detection of DNA modifications with high accuracy (24). Compared to traditional bisulfite conversion following short-read sequencing, the gold standard for DNA methylation detection, our approach offers many potential advantages. The method omits conventional DNA-damaging bisulfite treatment and PCR amplification, unlike short-read sequencing, long-read sequencing allows the mapping of repetitive or low-complexity regions (25). Importantly, a high correlation of methylation frequency data between nanopore sequencing at low depth and bisulfite sequencing has been reported (26, 27).

The function of DNA methylation is diverse and depends on the genomic regions where it occurs. DNA hypermethylation in the promoter region and 1–5 kb upstream of the transcription start site is associated with gene silencing (28). Hypermethylated sites with significant methylation difference (*p*<1.0x10^–10^) in these regions were annotated to the long-noncoding RNAs (lncRNAs) *LOC107984012* and *LOC105373526*, ribosomal *RNA5S6* and *RNA5S5*, the genes *ATG16L2, GRB10, CLUAP1* and C16orf90, and the pseudogene *MRPS11P1.* Only *GRB10* has been previously associated with diabetes-related complications. Polymorphism in *GRB10* has been associated with the risk of coronary heart disease in individuals with type 2 diabetes (29).

Hypomethylated CpG sites with significant methylation difference (*p*<1.0x10^–10^) in these regions were annotated to the lncRNAs *LINC00355, KCNK15-AS1, LOC101928797, LINC00654*, and *FLG-AS1, LOC100996664*, the microRNA mir-6080, the genes *CD1D, RGPD2, CDIN1*, *GXYLT1*, *PLAGL1*, and *CHRNE*, and the pseudogenes *ELL2P1*, and *CCDC144NL. CD1D* (30), *GXYLT1* (31)*, LINC00355* (32), *FLG-AS1* (33), *CCDC144NL* (34), and *PLAGL1* (35) have been previously associated with diabetes or diabetes-related complications. For example, loci near the *GXYLT1* gene have been associated with treatment-related reductions in HbA1c in type 2 diabetes (31). Overexpression of *FLG-AS1* has been shown to attenuate inflammation, oxidative stress, and apoptosis in retinal epithelial cells exposed to high glucose (33). Additionally, *CCDC144NL* was one of the genes selected for a combinatorial molecular signature as a potential biomarker for early detection of proliferative diabetic retinopathy (34).

Most of our findings are consistent with previously reported results. Several pathways, that have been significantly enriched in our study, have already been reported to be associated with diabetes-related complications. Phospholipase D signaling pathway (36), phosphatidylinositol signaling system (37), and retrograde endocannabinoid signaling (38), endocytosis (39), MAPK signaling pathway (40), cAMP signaling pathway (41), calcium signaling pathway (42), and AMPK signaling pathway (43) are involved in one or more mechanisms that cause diabetes-related complications, including inflammation, oxidative stress, apoptosis, and insulin resistance. Conversely, the Rap1 signaling pathway (44), oxytocin signaling pathway (45), and relaxin signaling pathway (46) have been shown to play a protective role in diabetes. Additionally, our analysis revealed enrichment in neurochemical synapses. Hyperglycemia is known to induce synaptic dysfunction and disrupt the balance of neurotransmitter secretion (47). Furthermore, differential gene expression analyses in various diabetic tissues were similar to our findings, with overlapping pathways observed in skeletal muscle (48), β-cell-enriched tissue (49), diabetic kidney (50, 51), and diabetic peripheral neuropathy (52).

Among the genes common to the identified signaling pathways, *ADCY4*, *GNAS*, *MAPK10*, *PIK3CD*, and *RAF1* had hypomethylated CpG sites in the promoter region and 1–5 kb upstream of the transcription start site, which may imply increase gene expression. ADCY4 (53) and GNAS (54) are both involved in the cAMP signaling pathway, which plays an important role in glucose metabolism (55), inflammation (41), and fibrosis (56), as well as in diabetic kidney disease (57), diabetic retinopathy (58), and neuropathy (59).

GNAS is important for β-cell insulin secretion, with reduced expression observed in pancreatic islets from individuals with type 2 diabetes (60) and in urine from individuals with diabetes (61). Downregulation of *ADCY4* has been associated to impaired mitochondrial function in murine hearts (62), while hyperglycemia-induced cardiac hypertrophy and apoptosis act through endoplasmic reticulum stress-JNK3 signaling pathway (63). JNK3 has been shown to play a critical role in diabetes-induced atrial fibrillation in mice (64).

Advanced glycated end products (AGEs) generated during prolonged hyperglycemia contribute to diabetes-related complications by promoting oxidative stress and inflammation. RAF1 activation by AGEs induces oxidative stress and inflammation in vascular endothelial cells, contributing to diabetic retinopathy (65). Similarly, PIK3CD plays a significant role in diabetic retinopathy, with high glucose levels upregulating its expression and promoting retinal angiogenesis, while its inhibition suppresses pathological angiogenesis (66, 67). Furthermore, decreased DNA methylation in the CpG site annotated to *PIK3CD* is identified in individuals with proliferative diabetic retinopathy (10).

Hypermethylated regions were located in the intron of *AL592295.3*, a long non-coding RNA, and in the promoter region of *AGGF1P1* and 1–5 kb upstream region of *RARRES2P4*. Both are processed pseudogenes; whose DNA sequences resemble functional genes. They cannot produce functional proteins; however, they may still have regulatory functions and thus play an important role in biological and pathological processes (68). The parental genes of these two pseudogenes are *AGGF1* and *RARRES2*. AGGF1 is an angiogenic factor and plays a critical role in vascular development. In diabetic mice, AGGF1 counteracts the damaging effect of hyperglycemia on endothelial progenitor cells (69). *RARRES2* encodes chemerin, an adipokine involved in adipogenesis. Chemerin has been shown to be a risk factor for the development of diabetic kidney disease (70).

Hypomethylated regions were located in exon 1 of the *GFD7* gene and in the promoter region of *LINC00355*. Hypomethylation in exon 1 of the *GDF7* gene was previously reported in an epigenome-wide association study in whole blood of Ghanaian participants with type 2 diabetes (71). In diabetic nephropathy *LINC00355* has been shown to be upregulated and to increase endoplasmic reticulum stress thus contributing to podocyte injury (32).

Prolonged hyperglycemia has long been associated with oxidative stress, AGEs, and inflammation (72). Interestingly, our results show that hyperglycemia directly affects DNA methylation before the onset of diabetes-related complications. We observed alterations in DNA methylation in genes and regions previously implicated in the pathogenesis of diabetes-related complications. Furthermore, we identified differentially methylated CpG sites in genes previously not linked to diabetes or diabetes-related complications. These includes non-coding RNAs, such as lncRNAs, microRNAs, and ribosomal RNAs. They play an important role in the regulation of biological processes and their aberrant expression has been observed in a wide range of human diseases, including in diabetes and its complications (73–75).

Several limitations need to be considered in our study. First, the sample of choice was the whole blood, which include diverse ratio of several different cell types with their own methylation profile. Furthermore, since DNA methylation can be tissue specific, our results do not necessarily reflect the methylation profile in other tissues that are directly affected in diabetes complications. Second, the participants’ DNA was pooled and sequenced as a single sample. Pooling of DNA samples is cost and time effective. However, it can introduce several potential biases including loss of individual variability, biological heterogeneity, and difficulty in data interpretation, due to the slight possibility that each participant is not equally represented in the pool. All precautions were taken to minimize these biases. Finally, we did not perform differential expression analysis, to see the effect of DNA methylation on gene expression.

## 5 Conclusions

Our study demonstrates that alterations in DNA methylation in blood samples from individuals with T1D precede the clinical manifestation of chronic complications. Differential methylation was identified in genes and pathways that have been previously associated with chronic diabetes complications, as well as in genes, non-coding RNAs, and pseudogenes not previously reported in the context of diabetes. As DNA methylation alterations after prolonged hyperglycemia persist also after the normalization of glycemic control, it could be potentially used as a biomarker for early detection and molecular phenotyping of individuals at an increased risk for diabetes-related complications. Furthermore, direct DNA sequencing methylation studies with long-read nanopore sequencing have become more commercially available and the data interpretation has improved in accuracy and efficiency with advances in bioinformatic tools and algorithms. This could enable individually tailored clinical diagnostics and interventions. Future research of DNA methylation in individuals could explore the specific mechanisms underlying these observed DNA methylation alterations and provide valuable insights into the pathogenesis of diabetes-related complications.

## Data availability statement

The datasets presented in this study can be found in online repositories. The name of the repository and accession number can be found here: https://www.ncbi.nlm.nih.gov/bioproject, accession number PRJNA1117600.

## Ethics statement

The studies involving humans were approved by Committee for Medical Ethics of the Republic of Slovenia. The studies were conducted in accordance with the local legislation and institutional requirements. Written informed consent for participation in this study was provided by the participants’ legal guardians/next of kin.

## Author contributions

BČK - Writing – original draft, Formal analysis, Visualization, Data curation. JK - Writing – review & editing, Conceptualization, Supervisio. RŠ - Writing – review & editing, Supervision. TT - Writing – review & editing, Supervision. BJB - Writing – review & editing, Data curation, Supervision. JG - Writing – review & editing, Investigation. TB - Writing – review & editing, Conceptualization, Resources. NB - Writing – review & editing, Funding acquisition, Resources. KD - Writing – review & editing, Investigation, Resources, Project administration.
